# Vertebroplasty with posterior spinal fusion for osteoporotic vertebral fracture using computer-assisted rod contouring system: A new minimally invasive technique

**DOI:** 10.1016/j.ijscr.2020.06.009

**Published:** 2020-06-12

**Authors:** Haruki Funao, Norihiro Isogai, Yutaka Sasao, Makoto Nishiyama, Ken Ishii

**Affiliations:** a Department of Orthopaedic Surgery, School of Medicine, International University of Health and Welfare, Japan; b Spine and Spinal Cord Center, International University of Health and Welfare Mita Hospital, Japan

**Keywords:** Osteoporotic vertebral fracture, Systemic scleroderma, Minimally invasive spine surgery, Vertebroplasty, Percutaneous pedicle screw, Computer-assisted rod contouring system

## Abstract

**Introduction:**

Surgical treatment of osteoporotic vertebral fracture (OVF) has been challenging for spine surgeons, because there are potential risks of instrumentation failure; such as screw loosening, loss of correction, or pseudarthrosis, due to bone fragility in elderly patients with several comorbidities.

**Presentation of case:**

A 68-year-old female presented with a severe low back pain and bilateral thigh pain. She had a history of systemic scleroderma, which was complicated by interstitial lung disease. Although she initially underwent non-surgical treatment with bracing for 7 months, her symptoms had progressively deteriorated, and her radiographs showed non-union at L1 and progressive kyphotic deformity at the thoracolumbar spine. Because an anterior approach was inadvisable due to interstitial lung disease, vertebroplasty with posterior spinal fusion was performed using percutaneous pedicle screws (PPS) at the upper most and lowest instrumented vertebra combined with sublaminar taping and computer-assisted rod contouring system. Good bony union was achieved with no screw loosening at 1-year follow-up.

**Discussion:**

Various surgical procedures have been applied according to the fracture type or medical condition of the patient. Minimally invasive posterior spinal fusion would be a less invasive approach in patients with poor medical condition. PPS can prevent the excessive dissection of paravertebral muscles, and this is especially advantageous at the proximal and distal end of long constructs. A recent computer-assisted rod contouring system accurately matches each screw head resulting in reduced strength of the screw-bone interface.

**Conclusion:**

This technique would be beneficial in the elderly or immunocompromised patients with OVF.

## Highlights

• Surgical treatment of osteoporotic vertebral fracture (OVF) is challenging.
• A new minimally invasive technique of posterior spinal fusion was performed for OVF.
• This technique would be beneficial for elderly or immunocompromised patients.

## 1 Introduction

Osteoporotic vertebral fracture (OVF) is the most common bone fragility fracture and can occur after minor stress or trauma in patients with osteoporosis. OVF is associated with decreased HRQOL score [1] and delayed union and reduced activities of daily living (ADL) associated with cognitive decline [2]. Furthermore, patients with OVF have a higher mortality rate [3]. Nonsurgical treatment including medication or bracing is a first-line treatment for the majority of the patients with OVF [4]. However, surgical treatment is required when the failure of nonsurgical treatment results in pseudarthrosis, neurological deficits, or progressive kyphotic deformity.

Surgical treatment of OVF has been challenging for spine surgeons, because there are potential risks of instrumentation failure; such as screw loosening, loss of correction, or pseudarthrosis, due to bone fragility in elderly patients with several comorbidities. To date, various surgical procedures have been reported, including vertebroplasty or balloon kyphoplasty [5,6], anterior spinal fusion [7], posterior spinal fusion [8], vertebroplasty with posterior spinal fusion [9], posterior spinal shortening [10], vertebral column resection [11], and combined anterior and posterior fusion [12]. Needless to say, surgical invasiveness should be reduced in elderly patients with poor medical condition.

Here, we describe a new minimally invasive technique of vertebroplasty with posterior spinal fusion for OVF using PPS at the upper most and lowest instrumented vertebra (UIV, LIV) combined with sublaminar polyethylene taping and computer-assisted rod contouring system.

## 2 Case presentation

**Fig. 1:**
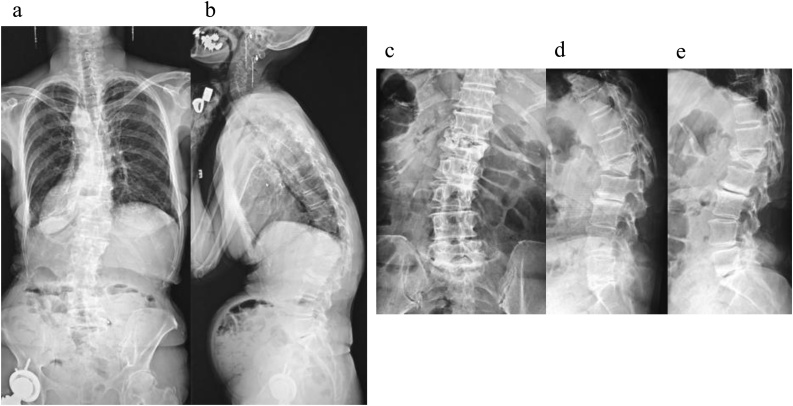
Preoperative radiographs of a 68-year-old female patient with OVF. Preoperative radiographs of a 68-year-old female showing kyphoscoliotic deformity at the thoracolumbar spine associated with non-union of OVF at L1. (a) Standing whole-spinopelvic AP radiograph, (b) Standing whole-spinopelvic lateral radiograph, (c) Lumbar AP radiograph, (d) Lumbar lateral radiograph in flexion, and (e) Lumbar lateral radiograph in extension.

**Fig. 2:**
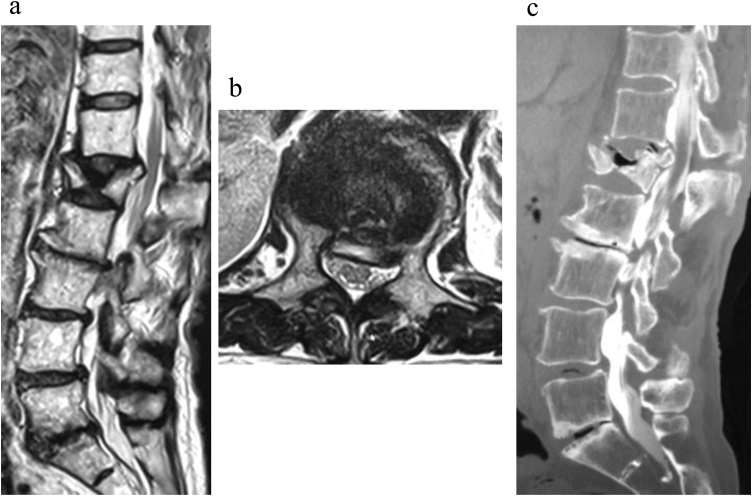
MRI and CT myelogram of the thoracolumbar spine. Views: (a) MRI of T2-weighted sagittal image, (b) MRI of T2-weighted axial image at L1, and (c) Sagittal image reconstruction of CT myelogram.

**Fig. 3:**
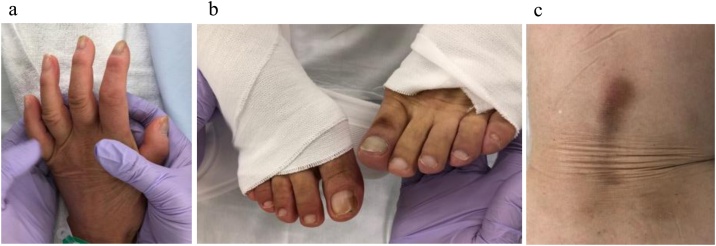
Clinical appearance of the patient with OVF who had a history of systemic scleroderma. The patient had a history of systemic scleroderma, which was complicated by interstitial lung disease. (a,b) Her fingers and toes were cold due to Raynaud’s phenomenon of systemic scleroderma. (c) Pressure sores were observed around the prominent L1 spinous process.

**Fig. 4:**
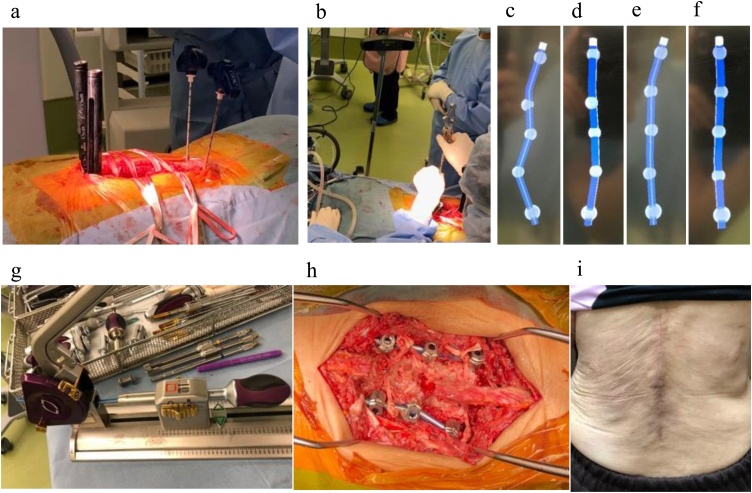
Intraoperative images. Minimally invasive technique of vertebroplasty with posterior spinal fusion combined with PPS, sublaminar polyethylene taping, and computer-assisted rod contouring was performed for OVF at L1. (a) Jamshidi needles were placed through lumbodorsal fascia, and the PPSs were placed at Th11 and L3 through guide wires under fluoroscopic guidance. (b) Each location for the screw heads were registered by the Bendini® Digitizer. (c–f) The Bendini® software enabled the estimation of rod lengths and visualization of the ideal rod contours; (c) Coronal rod contour on the left, (d) Sagittal rod contour on the left, (e) Coronal rod contour on the right, (f) Sagittal rod contour on the right. (g) A rod was placed into the multi-axial rod bender and was bent according to the instructions of the Bendini® software. (h) Sublaminar polyethylene tapes were tightened to the rods. (i) Postoperative surgical wound.

**Fig. 5:**
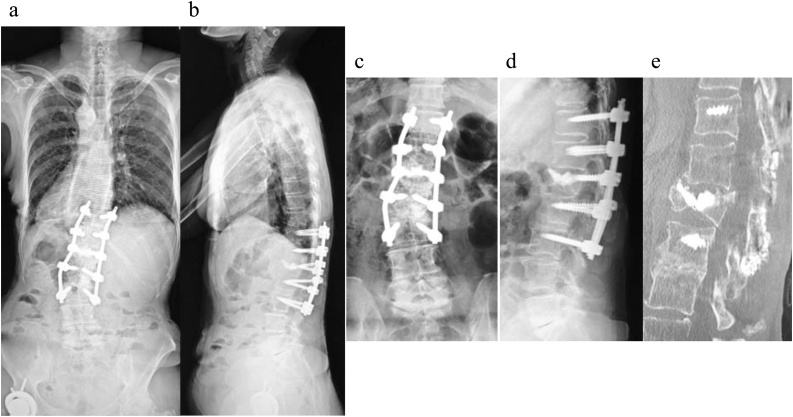
Postoperative radiographs and CT images at 1-year postoperatively. One-year postoperative radiographs: (a) Standing whole spinopelvic AP radiograph, (b) Standing whole-spinopelvic lateral radiograph, (c) Lumbar AP radiograph, (d) Lumbar lateral radiograph. (e) Sagittal image reconstruction of CT images at 1-year postoperatively.

A 68-year-old female presented with a severe low back pain and bilateral thigh pain due to L1 vertebral fracture. She had a history of left total hip arthroplasty, and systemic scleroderma which was complicated by interstitial lung disease. She was diagnosed with systemic scleroderma 20 years prior, and her recent dosage of oral steroid was 3 mg per day. Although she initially underwent bracing and daily administered parathyroid hormone for 7 months, her symptoms had progressively deteriorated. Her radiographs showed non-union at L1, kyphotic deformity of the thoracolumbar spine, and sagittal imbalance (Fig. 1a–e). Her lateral whole-spine radiograph showed; lumbar lordosis (Th12-S1) was 16°, local kyphosis (Th10-L2) was 37°, and sagittal vertical axis was 12.5 cm. Magnetic resonance imaging (MRI) of the lumbar spine showed a collapsed L1 vertebral body, and the posterior bone fragment compressing the spinal cord with bending motion (Fig. 2a,b). Her computed tomography (CT) myelogram showed an intravertebral vacuum cleft sign and destructive change of the L1 vertebral body (Fig. 2c). Her fingers and toes were cold due to Raynaud’s phenomenon of systemic scleroderma (Fig. 3a,b), and pressure sores were observed around the prominent L1 spinous process (Fig. 3c). Anterior spinal fusion with thoracotomy should be avoided to minimize postoperative pulmonary dysfunction due to interstitial lung disease. Given her condition, a vertebroplasty with posterior spinal fusion was performed using PPS at UIV and LIV combined with sublaminar polyethylene taping and in-situ fusion using a computer-assisted rod contouring system (Bendini®; NuVasive, Inc., San Diego, CA) (Fig. 4a–i). The operation time was 203 min with an estimated blood loss of 276 ml. The patients tolerated the procedure well with no major complications. Her symptoms markedly improved; her back pain was reduced to 1/10 in NRS and she denied any thigh pain. She could walk long distances without a T-cane 1-year postoperatively. Local kyphosis and sagittal imbalance were markedly restored; lumbar lordosis was 33°, local kyphosis was 14°, and sagittal vertical axis was 6.8 cm on her postoperative radiograph (Fig. 5a–d). Her postoperative CT showed good bony union without screw loosening or screw pull-out 1-year postoperatively (Fig. 5e). Although the scoliotic curve remained, she did not complain of related symptoms.

This case report follows the Surgical Case Report (SCARE) Guidelines 2018 [13].

### 2.1 Surgical technique

The patient was placed in a prone position, and a midline incision was made to allow a vertebroplasty at L1, insertions of pedicle screws at Th12, L1, and L2, placement of sublaminar polyethylene taping (Nesplon; Alfresa, Inc., Osaka, Japan) at Th12 and L2, and resection of prominent L1 spinous process for bone graft. A vertebroplasty was performed using hydroxyapatite (HA stick; Pentax, Co., Ltd., Tokyo, Japan). Laminectomy was not required, because the posterior bone fragment did not compress the spinal cord with stabilization, and laminae were important for the posterior fusion bed. Jamshidi needles were placed through the lumbodorsal fascia at Th11 (UIV) and L3 (LIV) under fluoroscopic guidance. One-shot imaging was used rather than continuous fluoroscopy to reduce radiation exposure [14]. A percutaneous guide wire (S-wire) was used to prevent the anterior migration of the guide wires [15]. The PPSs were placed at Th11 and L3 after tapping the pedicle holes through the guide wires (Fig. 4a). After the placement of pedicle screws, each location for the screw heads were registered by the Bendini® Digitizer (Fig. 4b). The Bendini® software enabled us to estimate the rod lengths and to identify the ideal rod contours (Fig. 4c–f). A rod was placed into the multi-axial rod bender (Fig. 4g) and was bent according to the instructions of the Bendini® software. The computer-assisted pre-bent rod was easily placed at both the PPS and pedicle screws, and the set screws were also smoothly tightened without screw pull-out. Sublaminar polyethylene tapes were tightened to the rods (Fig. 4h). After ample irrigation, local bone graft and β-tricalcium phosphate (Cerarebone; NGK Spark Plug Co., Ltd., Aichi, Japan) were placed on the decorticated lamina and facet joints. Her surgical wound healed well without any wound dehiscence (Fig. 4i).

### 2.2 Research registration

We register our surgical technique at http://www.researchregistry.com. The Unique Identifying Number from the Research Registry of this study is researchregistry5677.

## 3 Discussion

In the coming decades, clinicians will be required to manage an increasing number of patients with osteoporosis due to an aging population. Since the surgical treatment of OVF has been challenging in elderly patients with bone fragility or multiple comorbidities, surgical invasiveness should be reduced to prevent instrumentation failure and complications. To date, various surgical procedures have been applied according to the fracture type or medical condition of the patient: vertebroplasty or balloon kyphoplasty [5,6], anterior spinal fusion [7], posterior spinal fusion [8], vertebroplasty with posterior spinal fusion [9], posterior spinal shortening [10], vertebral column resection [11], and combined anterior and posterior fusion [12]. Although an anterior spinal fusion is advantageous to patients with OVF in providing a stable anterior column support, an anterior approach with thoracotomy for OVF at the thoracolumbar spine may be an invasive procedure. Therefore, the procedure is sometimes inadvisable in the elderly or immunocompromised patients with pulmonary dysfunction. Kashii et al. [16] reported that an equivalent improvement of neurological deficits and ADL function were achieved in anterior spinal fusion, posterior spinal shortening, and vertebroplasty with posterior spinal fusion. They reported that vertebroplasty with posterior spinal fusion showed the least surgical invasion.

Minimally invasive spinal fusion using PPS can be a less invasive posterior approach in patients with poor medical condition [17]. PPS can prevent the excessive dissection of paravertebral muscles, and this is especially advantageous at UIV and LIV to reduce the risk of proximal and distal junctional failure. However, pedicle screw fixation alone may result in screw loosening due to bone fragility; therefore, augmentation for screws such as hooks or sublaminar taping should be considered to reduce screw loosening or pull-out in patients with bone fragility [18]. Moreover, one of the drawbacks of PPS is the difficulty in estimating rod lengths and adequate rod contouring in multi-level spinal fusion. Manual rod bending can lead to inadequate rod bending, which can result in screw loosening or pull-out [19]. Recent techniques in computer-assisted rod bending [20] can provide an alternative rod contouring technique for multi-level PPSs with long rod constructs, because it enables us to estimate accurate rod lengths and contours. The computer-assisted pre-bent rod accurately matches each screw head; therefore, its precise placement would result in reduced strength of the screw-bone interface.

## 4 Conclusion

In conclusions, we describe a new minimally invasive technique of vertebroplasty with posterior spinal fusion combined with PPS, sublaminar polyethylene taping, and computer-assisted rod contouring system for OVF in an elderly patient with systemic scleroderma. Good bony union was achieved with no screw loosening and screw pull-out through the final follow-up. This technique would be especially beneficial for elderly or immunocompromised patients with OVF. Further investigation will be required to determine whether this technique is effective with a low complication rate for broader clinical use.

## Declaration of Competing Interest

We have no potential conflict of interest with regards to this article.

## Sources of funding

No funds were received in support of this work.

## Ethical approval

Not applicable. A single case report is exempt from ethical approval in our institution.

## Consent

Written informed consent was obtained from the patient for publication of this report and the accompanying images.

## Author contribution

Haruki Funao was a main surgeon, and Yutaka Sasao was an assisting surgeon of this surgery. Haruki Funao: design of the surgical procedure, draft of the manuscript with figures. Norihiro Isogai, Yutaka Sasao, Makoto Nishiyama, and Ken Ishii: substantial contributions to study design and data acquisition. All authors read and approved the final manuscript. Haruki Funao: final approval of the version to be published.

## Registration of research studies

The Unique Identifying Number from the Research Registry is researchregistry5677.

## Guarantor

Haruki Funao, M.D., Ph.D.

## Provenance and peer review

Not commissioned, externally peer-reviewed.

## References

[1] Oleksik A., Lips P., Dawson A. (2000). Health-related quality of life in postmenopausal women with low BMD with or without prevalent vertebral fractures. J. Bone Miner. Res..

[2] Takahashi S., Hoshino M., Tsujio T., Terai H., Suzuki A., Namikawa T., Kato M., Matsumura A., Takayama K., Nakamura H. (2017). Risk factors for cognitive decline following osteoporotic vertebral fractures: a multicenter cohort study. J. Orthop. Sci..

[3] Cooper C., Atkinson E.J., Jacobsen S.J., O’Fallon W.M., Melton L.J. (1993). Population-based study of survival after osteoporotic fractures. Am. J. Epidemiol..

[4] Lee H.M., Park S.Y., Lee S.H. (2012). Comparative analysis of clinical outcomes in patients with osteoporotic vertebral compression fractures (OVCFs): conservative treatment versus balloon kyphoplasty. Spine J..

[5] Galibert P., Deramond H., Rosat P., Le Gars D. (1987). Preliminary note on the treatment of vertebral angioma as well as painful and debilitating diseases. Neurochirurgie.

[6] Hardouin P., Fayada P., Leclet H., Kyphoplasty Chopin D. (2002). Joint Bone Spine.

[7] Kaneda K., Asano S., Hashimoto T., Satoh S., Fujiya M. (1992). The treatment of osteoporotic-posttraumatic vertebral collapse using the Kaneda device and a bioactive ceramic vertebral prosthesis. Spine (Phila Pa 1976).

[8] Ataka H., Tanno T., Yamazaki M. (2009). Posterior instrumented fusion without neural decompression for incomplete neurological deficits following vertebral collapse in the osteoporotic thoracolumbar spine. Eur. Spine J..

[9] Matsuyama Y., Goto M., Yoshihara H. (2004). Vertebral reconstruction with biodegradable calcium phosphate cement in the treatment of osteoporotic vertebral compression fracture using instrumentation. J. Spinal Disord. Tech..

[10] Saita K., Hoshino Y., Kikkawa I. (2000). Posterior spinal shortening for paraplegia after vertebral collapse caused by osteoporosis. Spine (Phila Pa 1976).

[11] Krishnakumar R., Lenke L.G. (2015). “Sternum-into-abdomen” deformity with abdominal compression following osteoporotic vertebral compression fractures managed by 2-level vertebral column resection and reconstruction. Spine.

[12] Nakashima H., Imagama S., Yukawa Y., Kanemura T., Kamiya M., Deguchi M. (2015). Comparative study of 2 surgical procedures for osteoporotic delayed vertebral collapse: anterior and posterior combined surgery versus posterior spinal fusion with vertebroplasty. Spine (Phila Pa 1976).

[13] Agha R.A., Borrelli M.R., Farwana R., Koshy K., Fowler A., Orgill D.P., For the SCARE Group (2018). The SCARE 2018 statement: updating consensus Surgical CAse REport (SCARE) guidelines. Int. J. Surg..

[14] Funao H., Ishii K., Momoshima S., Iwanami A., Hosogane N., Watanabe K., Nakamura M., Toyama Y., Matsumoto M. (2014). Surgeons’ exposure to radiation in single- and multi-level minimally invasive transforaminal lumbar interbody fusion; a prospective study. PLoS One.

[15] Ishii K., Kaneko Y., Funao H., Ishihara S., Shinohara A., Nakanishi K. (2015). A novel percutaneous guide wire (s-wire) for percutaneous pedicle screw insertion: its development, efficacy, and safety. Surg. Innov..

[16] Kashii M., Yamazaki R., Yamashita T. (2013). Surgical treatment for osteoporotic vertebral collapse with neurological deficits: retrospective comparative study of three procedures—anterior surgery versus posterior spinal shorting osteotomy versus posterior spinal fusion using vertebroplasty. Eur. Spine J..

[17] Kakarla U.K., Little A.S., Chang S.W., Sonntag V.K., Theodore N. (2010). Placement of percutaneous thoracic pedicle screws using neuronavigation. World Neurosurg..

[18] Hamasaki T., Tanaka N., Kim J., Okada M., Ochi M., Hutton W.C. (2010). Pedicle screw augmentation with polyethylene tape: a biomechanical study in the osteoporotic thoracolumbar spine. J. Spinal Disord. Tech..

[19] Paik H., Kang D.G., Lehman R.A. (2013). The biomechanical consequences of rod reduction on pedicle screws: should it be avoided?. Spine J..

[20] Tohmeh A., Isaacs R.E., Dooley Z.A., Turner A.W. (2014). Long construct pedicle screw reduction and residual forces are decreased using a computer-assisted spinal rod bending system. J. Spine Neurosurg..

